# The Prognosis of Primary Percutaneous Coronary Intervention after One Year Clinical Follow Up

**Published:** 2013-03-15

**Authors:** Yahya Dadjoo, Yadallah Mahmoodi

**Affiliations:** 1 Cardiovascular Research Center, Bagheiatallah University of Medical Sciences, Tehran, IR Iran

**Keywords:** Myocardial Infarction, Intervention, Mortality

## Abstract

**Objectives:**

The aim of this study was to evaluate the clinical outcomes, one year after primary percutaneous coronary intervention(PCI).

**Patients and Methods:**

From September 2009 to March 2012, primary PCI was performed on 70 cases, and the data relating to their catheterization were recorded. Peri-interventional treatment data included PCI with drug-eluting or bare-metal stent or balloon angioplasty alone.

**Results:**

The mean age of the patients was 61.34+11.31 years, and 72.9% of them were males. The ratios of patients with diabetes, hypertension and, hyperlipidemia were 61.4%, 71.4%, and 52.9% respectively.

In clinical follow-up, total incidence of death was 4.3%, with no death occurring during 30 days. However, 3 patients died after one-year, of which one patient (1.4%) had cardiac problem and the other 2 (2.9%) died because of non-cardiac reasons. Target vessel revascularization, reinfarction within 30 days, and mechanical complication or stroke were not found in any of the patients. Patients with hypertension (6%) and those with LAD ST-elevation myocardial infarction (5%) died after one year (*P*= 0.263 and *P*= 0.319 respectively). However, no mortality was reported in patients with RCA and LCX ST-elevation myocardial infarction. Of subjects with multivessel disease, 7% died after one-year (*P*= 0.161), but there was no reported mortality in those with single vessel disease.

**Conclusions:**

The prognosis was satisfactory in patients undergoing PCI after one year clinical follow up.

## 1. Introduction

Early reperfusion results in a reduced mortality rate and is the treatment of choice for ST-elevation myocardial infarction (STEMI) (1-3). Compared to fibrinolysis, primary percutaneous coronary intervention (PCI) is more effective in achieving patency of the infarct-related artery and reducing risk of mortality, re-infarction, and stroke (4). There is a critical link between early establishment of thrombolysis in myocardial infarction (TIMI)-3 flow with myocardial salvage and survival. Primary PCI attains TIMI-3 flow in more than 90% of patients (5). In contrast, less than 65% of patients receiving a fibrin specific lytic agent achieve this reperfusion benchmark (5). In a combined meta-analysis of the GUSTO-I and PAMI-I/II trials, primary PCI resulted in an 86% reduction in the risk of mechanical complications compared to patients undergoing thrombolysis (6). In a multivariate analysis of 1375 patients, treatment with primary PCI was independently associated with a lower risk of free wall rupture (7). Thus, primary PCI has become the preferred reperfusion strategy (4). Early administration of adjunctive antithrombotic therapy with acetylsalicylic acid (ASA) and heparin leads to improved initial patency of the infarct-related artery (8). Additional platelet inhibition with clopidogrel, improves outcome of patients with stable coronary artery disease undergoing PCI and of subjects with acute coronary syndromes undergoing PCI (9-12). Therefore, current guidelines recommend early initiation of antithrombotic therapy with ASA and heparin as well as an early loading dose clopidogrel in acute coronary syndromes (1-3). Similarly, the benefit of reperfusion therapy for STEMI is strongly dependent on the interval between onset of symptom and treatment (13, 14). To avoid unacceptable time delays, STEMI guidelines as well as recent overviews have outlined the importance of organized systems of care network in order to shorten the time delay from electrocardiogram (ECG) diagnosis, as the first medical contact, to the first balloon dilatation in an efficient catheter laboratory by experienced personnel (15-18). However, time delays are not always important for patients referred for primary PCI , and short and longer time delays have been shown to lead to similar mortality rates in mechanically reperfused patients (15, 19-21), while success of fibrinolytic therapy is always time dependent (11, 15, 16). The aim of the present study was to evaluate one year follow up of clinical outcomes in regard to 30 days and, one year mortality rates, 30 days re-infarction, target vessel revascularization, stroke and mechanical complications including acute mitral regurgitation, ventricular septal defect, and free wall rupture after primary PCI.

The aim of the present study was to evaluate one year follow up of clinical outcomes in regard to 30 days and, one year mortality rates, 30 days re-infarction, target vessel revascularization, stroke and mechanical complications including acute mitral regurgitation, ventricular septal defect, and free wall rupture after primary PCI.

## 2. Patients and Methods

Of all patients undergoing coronary angiography at our institution since September 2009 to march 2012, 70 patients underwent primary PCI. Data obtained from catheterization laboratories included demographic records, risk factors, prior coronary interventions or bypass surgery, previous myocardial infarction, complications during procedure, as well as episodes of cardiogenic shock or resuscitation before or during the interventional procedure of the index event. Peri-interventional treatment data covered PCI with drug-eluting or bare-metal stent or balloon angioplasty only, as well as TIMI flow before and after intervention and no reflow phenomena during the procedure. Additional data included adjunctive therapies with GP IIb/IIIa antagonists in the catheterization laboratory, or use of thrombus aspiration.

According to CARDS data standards(22), STEMI was diagnosed in the presence of persistent angina pectoris for at least 20 min and ST-segment elevation of >1mm in at least two standard leads or >2mm in at least two continuous pre-cordial leads, or presumably the presence of a new left bundle branch block (23).

The patients were evaluated regarding clinical outcomes including 30-days and one-year mortalities, 30 days reinfarction, target vessel revascularization, stroke and mechanical complication comprising acute mitral regurgitation, ventricular septal defect, free wall rupture occurring in one year.

The study was approved by Bagheiatallah University of medical sciences and ethical committee. After collecting the data, all the statistical analyses were carried out using SPSS version 17 via chi-square test, and P values of <0.05 were considered statistically significant*.*

## 3. Results

Of the 70 patients who underwent primary PCI, 51 (72.9%) were men and 19 (27.1%) were women, and 37 (52.9%) had hyperlipidemia. All 70 patients had DES (Resolute integrity and Biomatrix) and none had bare metal stent (BMS). Additionally, 17 patients (24.3%) were smokers and 53 (75.7%) were nonsmokers; 27(38.6%) and 43(61.4%) had single vessel and multivessel diseases respectively. Fifty (71.4%) patients had hypertension and 4 patients (5.7%) had a family history of myocardial infarction or ischemic heart disease with 66 (94.3%) subjects having no such history. Forty-three patients (61.4%) had DM and 27 (38.6) did not. No reflow phenomena did not develop in any of the patients during procedure. The percentage of LAD, RCA and LCX ST-elevation myocardial infarction (STEMI) was 57.1%, 34.3% and 8.6% respectively. Sixty –six patients (94.3) had adjunctive therapies with GP IIb/IIIa antagonists in the catheterization laboratory. Thrombus aspiration with aspiration catheter kit (ASAP) was used for 32 patients (45.7%) (Table 1).

**Table 1.** Clinical Characteristics for 70 Patients who Underwent Primory Percutoneous Coronary Intervention (PCI)

Variable	Primary PCI
Age (Years)	61.34±11.31
Sex (M:F)	51(72.9%): (%1.72)91
Hypertension (P:N)	50 (71.4%):20(28.6% )
Hyperlipidemia (P:N)	33(47.1%):(37(52.9%)
Smoker (P:N)	53(75.7%):(17(24.3%)
Family history (P:N)	66(94.3%):4(5.7%)
Drug eluting stent (P:N)	0(0%):70(100%)
Thrombus suction (P:N)	38(54.3%):32(45.7%)
Glycoprotein IIb/IIIa antagonists (P:N)	4(5.7%):66(94.3%)
Number of vessel (single:multiple)	43(61.4%):27(38.6%)
Type of vessel causing STEMI(LAD:RCA:LCX)	6(8.6%):24(34.3%):40(57.1%)

In clinical follow-up, total incidence of death was 4.3% (3 patients), and none of the patients died in 30-days. However, 3 patients (4.3%) died during the course of one year, of which 1 (1.4%) was due to cardiac problem and 2 (2.9%) because of non-cardiac problems. Target vessel revascularization, 30 days reinfarction, and mechanical complication or stroke did not develop in any of the patients. There was one-year mortality in 6 % (*P*= 0.263) and 5% (*P*=0.319) of the patients with hypertension and LAD ST-elevation myocardial infarction respectively. However, no mortality was reported in patients with RCA and LCX ST-elevation myocardial infarction. Although all patients with single vessel disease survived, 7% of those with multivessel disease died within one-year (*P*=0.161).

The result of this study showed satisfactory prognosis in that, there was no mortality in patients with target vessel revascularization, stroke and mechanical complication in the course of one year follow up, and low rate of mortality and re-infarction during this period. Maleness, hypertention, DM and no smoking may be the risk factors for acute STEMI. Hypertension (*P*=0.263), number of vessel stenosis during presentation of acute myocardial infarction considering single versus multi vessel stenosis (*P*=0.163), and type of vessel causing acute myocardial infarction including LAD, RCA or LCX were not significant predictors (*P*=0.319) of worsening clinical outcomes after primary PCI.

## 4. Discussion

Evidence favoring the primary angioplasty strategy derived from the primary coronary angioplasty trialists (PCAT), meta-analysis of 10 randomized trials revealed that primary PCI significantly reduced 30-day mortality, reinfarction and stroke (24). In a combined meta-analysis of the GUSTO-I and PAMI-I/II trials, primary PCI resulted in an 86% reduction in the risk of mechanical complications compared to patients undergoing thrombolysis (6). The main finding in the present study is that primary PCI, preferably with stenting, and the GP IIb/IIIa receptor antagonist were not associated with any mechanical complication, target vessel revascularization, 30 days reinfarction and stroke, along with a very low cardiac mortality rate. In this connection, there was no in 30 days and 4.3% mortality in one year of which 1.4% was due to cardiac problem and 2.9% was due to non-cardiac episode. The results of this study were consistent with those of the previous investigations (25,26), but rate of target vessel revascularization in our study was significantly lower. In our study, most of the patients who underwent primary PCI were males with hypertention and DM. However, the primary PCI was predominantly performed on non-smoking patients. This revealed that male, hypertention, DM and no smoking may be the risk factors of acute STEMI. Finally, in the present study, clinical outcomes was satisfactory and primary PCI is a viable choice for treatment of patients with ST-elevation myocardial infarction.
